# Factors influencing the 5-year survival rate of oral cancer patients in the Mongolian population: a retrospective cohort study

**DOI:** 10.3389/froh.2023.1292720

**Published:** 2023-12-15

**Authors:** Oyuntsetseg Davaatsend, Munkhdul Altannamar, Badral Batbayar, Urjinlkham Jagdagsuren

**Affiliations:** ^1^ Department of Maxilla-Facial Surgery School of Dentistry, Mongolian National University of Medical Sciences, Ulaanbaatar, Mongolia; ^2^ Department of Maxilla-Facial Surgery, School of Dentistry, Ach Medical University, Ulaanbaatar, Mongolia; ^3^ Department of Restorative Dentistry, School of Dentistry, Mongolian National University of Medical Sciences, Ulaanbaatar, Mongolia

**Keywords:** tongue cancer, survival rate, TNM stage, squamous cell carcinoma, prognosis

## Abstract

**Introduction:**

The high mortality rate of head and neck cancers, particularly oral cancer, poses a significant health challenge in developing nations such as Mongolia. This retrospective survival analysis study was conducted to identify factors influencing the 5-year survival rate of oral squamous cell carcinoma patients.

**Methods:**

The study analyzed data from 173 patients diagnosed with oral squamous cell carcinoma, including multiple variables such as age, gender, residence, education, tobacco and alcohol consumption, oral health indicators, family history, precancerous conditions, cancer characteristics, treatment, rehabilitation, cancer recurrence, and 5-year survival. Survival analysis was conducted using the Kaplan–Meier method, and STATA was used for statistical analysis.

**Results:**

The study revealed a 5-year survival rate of 50.3% for oral cancer patients, with a survival rate of 38% for tongue cancer patients. Age, residence, cancer stage, and cancer recurrence were identified as significant survival predictors. Compared to those aged 60 or younger, the hazard ratio (HR) for patients aged 61 or older was 1.52. Survival was associated with female gender (HR = 0.47, CI = 0.29–0.77). Urban residence was associated with decreased survival (HR = 1.92, CI = 1.22–3.05). Significantly worse survival was associated with the presence of cancer recurrence (HR = 1.99, CI = 1.15–3.04). Oral cancer patients in stage IV had a fourfold higher risk of mortality compared to those in stage I (HR = 4.08, CI = 1.2–13.84).

**Conclusion:**

This research highlights the influence of age, urban habitation, and cancer recurrence on oral cancer survival. Age, urban residence, and cancer recurrence were all associated with decreased survival, whereas cancer at stage IV substantially increased the risk of death. The significance of early detection, treatment, and active surveillance to identify oral cancer at an early stage is highlighted by these findings. Compared to industrialized nations, Mongolia's lower oral cancer survival rates emphasize the need to increase public awareness and education. A comprehensive approach is required to improve oral cancer patient survival rates and quality of life, including emphasizing early detection through active surveillance, implementing preventive measures, and advancing cancer education initiatives.

## Introduction

Oral cancer is disproportionately prevalent in low- and middle-income countries (LMICs), where the 5-year survival rate is low. In 2020, an estimated 476125 cases of oral or oropharyngeal cancer were diagnosed worldwide (1). According to the 2013–2019 SEER report on oral cavity and pharynx cancer, the relative 5-year survival rate was 68.5% (2). The prevalence of oral cancer differs considerably based on geographical location and population characteristics (3). South and Southeast Asia have the greatest rates of oral cancer incidence (4).

Oral cancer risk factors include tobacco use, alcohol consumption, diet, dental health, medical comorbidities, HPV infection, and behaviors such as betel nut chewing (1, 5–9). Non-smokers can also develop oral cavity cancer (10). Younger individuals are increasingly affected, notably those with tongue cancer (6). Based on a systematic evaluation conducted in Saudi Arabia, the prevalence of oral cancer ranged from 21.6% to 68.2%. The ratio of men to women varied from 36.6% to 65.4% (11). According to a Chinese study, oral cancer patients with a body mass index of less than 18.5 kg/m^2^, an age of less than 55 years, advanced clinical stages (II–IV), and weak differentiation had poorer survival outcomes (12). Low education, farming, and a low monthly household income were identified as significant risk factors for oral cancer in an Indian study (13).

In addition to socioeconomic factors, tumor characteristics such as stage, location, cell differentiation, type of treatment administered, and the quality of post-treatment care influence the overall survival rate (14–16). For instance, the 5-year survival rate for oral cancer patients after surgery varied significantly by pathological tumor, node, metastasis (TNM) stage, with stage I having the highest survival rate (90%) and stage IV having the lowest (45%), and the recurrence of cervical lymph node cancer had a significant negative effect on the survival rate (17). In another study, 51.1% of patients had tongue cancer, and 49.1% received postoperative radiotherapy; node-negative patients had a 5-year survival rate of 79% compared to 59% for node-positive patients (18). A study in the Netherlands revealed that 5-year relative survival decreased with increasing stages (19).

The primary outcome of our investigation was determining the 5-year survival rate of individuals diagnosed with oral cancer measured by histopathologic grade. Comprehensive research on the risk factors associated with oral cancer recurrence is scarce, a deficiency that must be addressed. It is essential to investigate these risk factors to enable early diagnosis, individualized treatment, and enhanced patient outcomes in Mongolia.

This retrospective survival analysis study aims to provide a comprehensive understanding of a variety of variables affecting oral cancer survival, such as demographic factors, lifestyle behaviors, clinical characteristics, treatment modalities, and other pertinent factors.

## Materials and methods

### Study design

Our study used medical records from 173 patients diagnosed with squamous cell carcinoma of the mouth at the National Cancer Center of Mongolia's Department of Head and Neck Surgery, Radio, and Chemotherapy between 2012 and 2017.

### Study setting, participants, and recruitment

Patients diagnosed with squamous cell carcinoma of the oral cavity who were eligible for the investigation were recruited at the National Cancer Center between 2012 and 2017. Inclusion criteria for the study included a verified diagnosis of oral cancer via biopsy, assuring correct disease identification. To focus entirely on the impact of oral cancer, the patients included had no history of malignant tumors in any other part of their body. Accessing and analyzing the medical data of eligible patients who satisfied these predefined criteria was part of the recruitment procedure. The research team periodically extracted information from the participants’ medical records regarding oral cancer survival and relevant risk factors. Healthcare providers used a standardized patient medical history form to ensure the collection of consistent and reliable data on variables of interest, such as gender, age, tumor site, histopathologic grade, stage, alcohol and cigarette use, combination therapy, and the presence of cervical lymph node metastases.

#### Exclusion criteria

Patients who met any of the following exclusion criteria were excluded from the study:

• Non-oral cancer-related causes of death: Individuals who died from causes unrelated to oral cancer were excluded from the study. This criterion ensured that the analysis focused primarily on survival outcomes related to oral cancer.
• Patients with a history of malignant tumors in body regions other than the oral cavity were excluded from the study. This exclusion contributed to preserving a homogeneous study population whose singular focus was oral cancer.

### Variables

The primary outcome of interest in our investigation was the 5-year survival rate of individuals with oral cancer. Initial tumor site (lips, tongue, gums, mouth floor, palate), histopathologic grade, cancer stage (as classified by the American Joint Committee on Cancer Guide TNM stage classification) (20) were used as secondary outcomes. Furthermore, we identified oral cancer subsites using the International Classification of Diseases for Oncology (ICD-10) categories, which included lips (C00), tongue (C02), gums (C03), mouth floor (C04), and palate (C05) (21). Our analysis predictor variables include a wide range of characteristics, including demographic factors such as age, gender, and place of residence, lifestyle decisions such as tobacco and alcohol intake, and clinical indicators such as tumor size, cancer stage, and treatment techniques. The degree of tumor cell differentiation was classified as G1 well-differentiated, G2 moderately differentiated, G3 poorly differentiated, and G4 undifferentiated (22, 23). Tumor recurrence was the recurrence of tumor cells during the follow-up period after tumor treatment. Since the outdated hospital registration system constrained medical data, cancer recurrence was quantified as a binary variable, “Yes” or “No”, also applying to tobacco and alcohol intake.

### Sample size

This retrospective cohort study sample size comprised 173 individuals with oral cancer. These participants were selected based on the availability of medical records and the inclusion criterion of having been diagnosed with squamous cell carcinoma of oral cancer at the National Cancer Center.

### Statistical analysis

Stata 15 was used for all statistical analyses. Some quantitative factors included age, tumor size, lymph node, and stage. These variables were classified to improve data analysis and interpretation. In the age variable, for example, the age range was separated into distinct categories, such as 21–30, 31–40, 41–50, and so on. Similarly, the tumor size variable was divided into T1, T2, T3, and T4 to describe distinct phases of tumor size. The lymph node variable was classified as N0, *N* > 1, or NX, depending on whether lymph nodes were included. The number of observations in each category is given.

The stage variable was divided into four cancer stages: I, II, III, and IV. Frequencies and percentages were used to represent categorical variables. We used the Kaplan–Meier method for survival analysis, and the log-rank test was used to examine the survival distribution across factors. To determine hazard ratios, Cox proportional-hazards regressions were performed on oral cancer patients. To examine oral cancer survival factors, univariate and multivariate Cox regression analyses were used. To assess independent risk variables for oral cancer recurrence, univariate and multivariate logistic regressions were performed, and odds ratios were calculated. All statistical tests were two-sided, and *P*-values less than 0.05 were considered significant. For all hazard ratios, we provided 95% confidence intervals. We chose complete case analysis as the strategy for dealing with missing data in the study. Complete case analysis entailed examining only cases with complete data and removing any missing variables.

### Ethical considerations

We followed strict ethical guidelines when performing our study to safeguard the participants’ safety and well-being. The Research Ethics Committee of the Mongolian National University of Medical Sciences authorized the research protocol. On June 8, 2021, the Research Ethics Committee of the Mongolian National University of Medical Sciences granted ethical approval to the research protocol (Approval No. 2021/3-07). Patient information was kept entirely confidential, and all data obtained was anonymized to preserve patient privacy. In addition, we scrupulously adhered to the criteria for conducting retrospective research and treated medical records with the utmost care and secrecy. Data extraction and analysis were carried out in accordance with data protection standards while respecting patient privacy.

## Results

**Figure 1:**
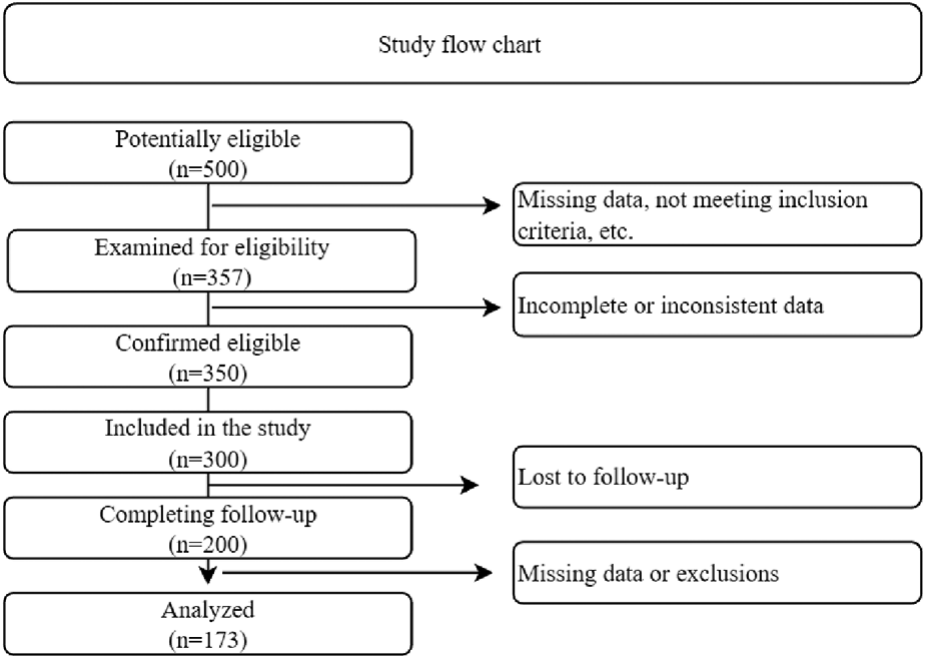
Study flow chart.

In the retrospective cohort design, the research began by identifying a pool of 500 potentially eligible individuals. After scrutinizing the eligibility of 143 individuals based on predefined criteria, it was determined that they were ineligible for various reasons, such as missing data or not meeting the inclusion criteria. This produced a confirmed cohort of 357 eligible individuals. Nonetheless, during the retrospective data collection process, some individuals were excluded due to insufficient or inconsistent data, resulting in a final cohort size of 300. One hundred participants were lost to follow-up, resulting in a sample size reduction to 200 participants who completed the entire follow-up period. This cohort of 200 individuals was then analyzed, considering any missing data or exclusions that occurred during the study. Finally, data analysis was performed on a subset of 173 individuals, which represented the study's final sample size, as show in Figure 1.

**Figure 2:**
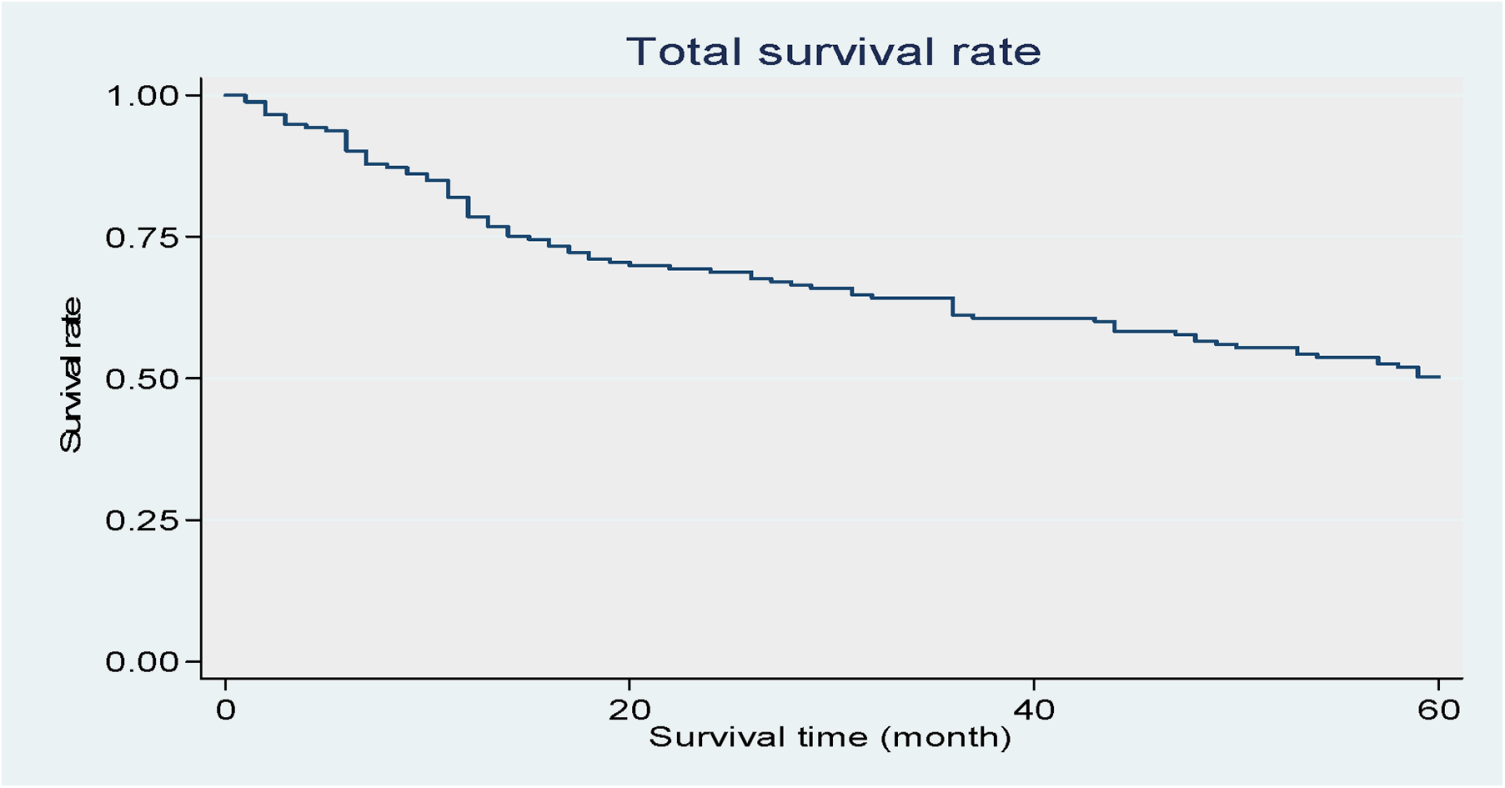
Shows that the total survival rate of the study's oral cancer patients decreased over time. Kaplan–Meier curve of the patients.

Figure 2 shows that the total survival rate of the study’s oral cancer patients decreased over time.

A total of 173 oral cancer cases were registered during the study period. Of these, 109 cases (63.0%) presented in males and 64 (37.0%) in females. Forty-nine patients (28.3%) were 61–70 years old, accounting for a significant proportion of all patients. The largest group in this retrospective cohort had an intermediate education level. Fifty-six percent of the patients lived in urban areas. Among the respondents, 97 were tobacco users (56.1%), 131 consumed alcohol (75.7%), and 156 had negative family histories (90.2%). Ten patients (5.8%) had leucoplakia with precancerous conditions. For almost half of the patients, the most common site of cancer was the tongue (79, 45.7%), followed by the lips (23, 13.3%) and hard and soft palate (16, 9.2%). At the time of diagnosis, 73 patients (42.2%) were in stage III, and 132 cases (76.3%) were well-differentiated. More than half of the study participants (110, 64%) underwent solo surgery. Of the remaining participants, 15 (8.7%) underwent surgery combined with chemotherapy, and 14 (8.1%) had surgery and radiotherapy. Cancer recurrence was presented in 26 patients (15%), as shown in Table 1.

**Table 1:** Socio-demographic characteristics, clinical characteristics, and treatment of oral cancer patients (*N* = 173).

Variables	Count (*N*)	Percent (%)
Age		
21–30	9	5.2
31–40	18	10.4
41–50	18	10.4
51–60	39	22.5
61–70	49	28.3
71–80	32	18.5
Over 81	8	4.6
Gender		
Male	109	63.0
Female	64	37.0
Residence		
Urban	76	43.9
Rural	97	56.1
Education		
None	5	2.9
Basic	26	15.0
Intermediate	81	46.8
Short-cycle tertiary	17	9.8
Advanced	44	25.4
Tobacco consumption		
No	97	56.1
Yes	76	43.9
Alcohol consumption		
No	131	75.7
Yes	42	24.3
Tobacco and alcohol use		
No	135	78.0
Yes	38	22.0
Chipped teeth		
No	158	91.3
Yes	15	8.7
Denture sores		
No	150	86.7
Yes	23	13.3
Family history		
No	156	90.2
Yes	17	9.8
Precancerous conditions		
No	152	87.9
Leukoplakia	10	5.8
Others	11	6.3
Cancer location		
Tongue	79	45.7
Lip	23	13.3
Cheek lining	10	5.8
Gums	15	8.7
Floor of the mouth	16	9.2
Hard palate	9	5.2
Soft palate	16	9.2
Retromolar space	5	2.9
Tumor size		
T1	21	12.1
T2	55	31.8
T3	56	32.4
T4	41	23.7
Lymph node		
N0	36	20.8
*N* > 1	115	66.5
NX	22	12.7
Metastasis		
M0	137	79.2
M1	4	2.3
MX	32	18.5
Stage		
I	11	6.4
II	25	14.5
III	73	42.2
IVA	47	27.2
IVB	13	7.5
IVC	4	2.3
Pathological grading		
G1 well-differentiated	132	76.3
G2 moderately differentiated	5	2.9
G3 poorly differentiated	31	17.9
G4 undifferentiated	5	2.9
Treatment		
Surgery	110	64.0
CT	5	2.9
RT	2	1.2
Surgery + CT	15	8.7
Surgery + RT	14	8.1
CT + RT	8	4.7
Surgery + CT + RT	18	10.5
Rehabilitation		
Yes	75	43.4
No	98	56.6
Cancer recurrence		
No	147	85.0
Yes	26	15.0
Survival in 5 years		
Alive	87	50.3
Passed away	86	49.7

CT, chemotherapy; RT, radiotherapy.

### Univariate analysis of predictive factors of five-year survival in oral cancer patients

The 5-year survival rates and univariate analysis of several prognostic variables were investigated in this study comprising 173 participants (Table 2). The study found that age had a significant impact on survival, with younger people (21–30 years) having the highest survival rate (77.8%) and lower hazard ratios, while older age groups had progressively lower survival rates and higher hazard ratios, with those over 81 years having the lowest survival (37.5%). Gender was also a factor, with females having a greater 5-year survival rate (67.2%) than males (40.4%). The effect of residence was minor, with rural participants surviving at a slightly higher rate (56.7%) than urban participants (42.1%). Tobacco use, alcohol use, and the presence of chipped teeth were all linked to a lower chance of survival, while education, denture sores, family history, and precancerous diseases had no effect.

**Table 2:** 5-year survival of study participants and univariate analysis of prognostic factors (*N* = 173).

	Total survival and in 5 years		*P*-value	Total survival (months) in 5 years	Log-rank P &	Hazard ratio (95% CI)
	Count (*N*)	Percent (%)		Median (min–max)		
Age						
21–30	7	77.8	0.082	60 (3–60)	0.051	1
31–40	14	77.8		60 (7–60)		0.86 (0.15–4.69)
41–50	10	55.6		60 (1–60)		2.02 (0.43–9.52)
51–60	19	48.7		59 (4–60)		2.38 (0.24–0.56)
61–70	20	40.8		31 (1–60)		3.51 (0.84–14.74)
71–80	14	43.8		53 (9–60)		2.71 (0.63–11.69)
Over 81	3	37.5		22 (2–60)		4.12 (0.79–21.25)
Gender						
Male	44	40.4	0.001	44 (1–60)	0.002	1
Female	43	67.2		60 (1–60)		0.47 (0.29–0.77)
Residence						
Rural	55	56.7	0.057	60 (2–60)	0.042	1
Urban	32	42.1		48 (1–60)		1.54 (1.01–2.35)
Education						
Advanced	22	50.0	0.830	59.5 (1–60)	0.841	1
None	2	40.0		49 (6–60)		1.18 (0.35–3.95)
Basic	12	46.2		47.5 (10–60)		1.10 (0.56–2.15)
Intermediate	44	54.3		60 (1–60)		0.88 (0.52–1.50)
Short-cycle tertiary	7	41.2		59.5 (1–60)		1.28 (0.61–2.72)
Tobacco consumption						
No	56	57.7	0.027	60 (1–60)	0.071	1
Yes	31	40.8		46 (2–60)		1.47 (0.96–2.24)
Alcohol consumption						
No	71	54.2	0.069	60 (1–60)	0.087	1
Yes	16	38.1		44 (3–60)		1.49 (0.94–2.36)
Chipped teeth						
No	83	52.5	0.056	60 (1–60)	0.087	1
Yes	4	26.7		37 (6–60)		1.72 (0.91–3.24)
Denture sores						
No	77	51.3	0.483	60 (1–60)	0.431	1
Yes	10	43.5		47 (5–60)		1.26 (0.70–2.28)
Family history						
No	80	51.3	0.429	60 (1–60)	0.286	1
Yes	7	41.2		47 (5–60)		1.42 (0.74–2.75)
Precancerous conditions						
No	76	50.0	0.393	59.5 (1–60)	0.650	1
Leukoplakia	4	40.0		50 (3–60)		1.23 (0.53–2.82)
Others	7	70.0		60 (1–60)		0.54 (0.17–1.72)

Table 3 provides a comprehensive analysis of the 5-year survival rates and univariate prognostic factors of 173 Mongolians diagnosed with oral cancer. Notably, the location of the oral cavity malignancy had a substantial effect on survival. With a diminished 5-year survival rate of 38.0% and an associated HR of 3.81 (95% CI: 0.71–15.71), the risk of tongue cancer is elevated. Larger tumors (T3 and T4) were associated with decreased survival rates and higher HRs in comparison to smaller tumors (T1). Survival was substantially affected by lymph node involvement (N stage) and the presence of metastasis (M stage), with HRs indicating an increased risk with lymph node involvement and the presence of metastasis. Cancer stage significantly affected survival, with advanced stages (Stages III and IV) exhibiting decreased survival rates and higher HRs. In addition, the analysis considered the type of treatment, the presence of cancer recurrence, and the histopathologic grading of tumors. The HR of 2.78 [95% confidence interval (CI): 1.69–4.75] indicates that the presence of cancer recurrence significantly decreased survival.

**Table 3:** 5-year survival of study participants and univariate analysis of prognostic factors (*N* = 173).

	Total survival and in 5 years		*P*-value^a^	Total survival (months) in 5 years	Log-rank P &	Hazard ratio^b^ (95% CI)
	Count (*N*)	Percent (%)		Median (min–max)		
Cancer location						
Hard palate	7	77.8	0.155	60 (2–60)	0.130	1
Tongue	30	38.0		36 (2–60)		3.81 (0.93–15.71)
Lip	15	65.2		60 (4–60)		1.67 (0.35–7.88)
Cheek lining	6	60.0		60 (14–60)		1.84 (0.34–10.08)
Gums	8	53.3		60 (1–60)		2.78 (0.58–13.41)
Floor of the mouth	9	56.3		60 (1–60)		2.32 (0.48–11.18)
Soft palate	9	56.3		60 (5–60)		2.25 (0.47–10.83)
Retromolar space	3	60.0		60 (26–60)		1.82 (0.26–12.95)
Tumor size						
T1	13	61.9	0.001	60 (2–60)	<0.001	1
T2	37	67.3		60 (3–60)		0.89 (0.39–2.05)
T3	25	44.6		52.5 (2–60)		1.77 (0.81–3.85)
T4	12	29.3		26 (1–60)		2.88 (1.31–6.31)
Lymph node						
N0	28	77.8	<0.001	60 (6–60)	<0.001	1
*N* > 1	46	40.0		7.5 (2–18)		3.64 (1.74–7.57)
NX	13	59.1		60 (6–60)		2.01 (0.78–5.22)
Metastasis						
M0	73	53.3		60 (1–60)	<0.001	1
M1	0	0.0	0.079	7.5 (2–18)		7.29 (2.59–20.52)
MX	14	43.8		49 (3–60)		1.27 (0.75–2.14)
Stage						
I	8	72.7	<0.001	60 (8–60)	<0.001	1
II	20	80.0		60 (12–60)		0.74 (0.18–3.09)
III	41	56.2		60 (2–60)		1.83 (0.56–5.97)
IV	18	28.1		20.5 (1–60)		4.41 (1.37–14.23)
Pathological grading						
Well	64	48.5	0.848	58.5 (1–60)	0.943	1
Moderate	3	60.0		60 (7–60)		0.78 (0.19–3.19)
Poor	17	54.8		60 (6–60)		0.87 (0.49–1.55)
Undifferentiated	3	60.0		60 (4–60)		0.81 (0.19–3.30)
Treatment						
Surgery	63	57.3	0.074	60 (1–60)	<0.001	1
CT	1	20.0		16 (7–60)		3.16 (1.13–8.82)
RT	0	0.0		7.5 (6–9)		11.45 (2.64–49.68)
Surgery + CT	8	53.3		60 (2–60)		1.02 (0.46–2.27)
Surgery + RT	3	21.4		13 (2–60)		3.19 (1.64–6.19)
CT + RT	3	37.5		39.5 (6–60)		1.79 (0.71–4.51)
Surgery + CT + RT	8	44.4		40.5 (1–60)		1.59 (0.81–3.16)
Rehabilitation						
Yes	39	52.0	0.694	60 (1–60)	0.706	1
No	48	49.0		59 (1–60)		1.08 (0.71–1.66)
Cancer recurrence						
No	82	55.8	0.001	60 (1–60)	<0.001	1
Yes	5	19.2		16.5 (1–60)		2.78 (1.69–4.75)
Total	87	50.3		60 (1–60)		

^a^ Chi-square test, & long rank Mantel–Cox test (mean ± standard error), ^b^Cox regression.

### Multivariate analysis of predictive factors of 5-year survival in oral cancer patients

Table 4 provides unadjusted and confounder-adjusted survival estimates (where applicable) for the prognostic factors associated with oral cancer survival. The HR indicates the relative mortality risk associated with each factor. The confidence intervals (CI) at 95% demonstrate the precision of the estimates. In the analysis, adjustments were made for residence, cancer stage, surgery, and cancer recurrence. These confounding variables were included as they are known or hypothesized to be associated with oral cancer survival and could potentially confound the relationships between the other variables and survival outcomes. We completed multivariate Cox proportional hazards logistic regression, which included all significant prognostic factors from the univariate Cox regression model. Patients living in urban areas (HR = 1.92 CI = 1.21–3.05) were associated with poorer survival than those in rural areas. The presence of cancer recurrence (HR = 1.99 CI = 1.15–3.44) also significantly correlated with worse survival. Patients diagnosed in stage IV (HR = 4.08 CI = 1.2–13.84) had a four times higher risk of death related to oral cancer than patients in stage I. Age, pathological grade, and surgery did not statistically correlate with overall survival, as shown in Table 4.

**Table 4:** Results of multivariate analysis of prognostic factors for survival in oral cancer (*N* = 173).

Variable	Overall survival		
	HR^b^	95% CI	*P* ^a^
Age			
≤60 years	1		
>61 years	1.52	0.96–2.39	0.070
Residence			
Rural	1		
Urban	1.92	1.21–3.05	0.006
Stage			
I	1		
II	0.86	0.20–3.65	0.839
III	1.76	0.52–5.91	0.363
IV	4.08	1.20–13.84	0.024
Pathological grade			
Well-moderate	1		
Poor-undifferentiated	1.10	0.62–1.96	0.743
Surgery			
Yes	1		
No	0.83	0.49–1.39	0.486
Cancer recurrence			
No	1		
Yes	1.99	1.15–3.44	0.014

^a^ Chi-square test, ^b^Cox proportional hazards logistic regression, adjusted for all variables.

## Discussion

These results imply that age, place of residence, cancer stage, and cancer recurrence are significant survival predictors for oral cancer. A higher mortality risk is associated with advanced age, urban living, advanced cancer stage (IV), and cancer recurrence. Our study found that advanced age is significantly associated with poor survival, consistent with previous studies (24–26). Another substantial risk factor associated with low survival was living in a city. However, we cannot locate any previous studies that confirm our conclusion, which may be explained by the higher proportion of Mongolians living in Ulaanbaatar than in rural areas, as well as in part by screening accessibility.

Using the Kaplan–Meier method to calculate survival, our study revealed a 50.3% oral cancer survival rate. According to the 2013–2019 SEER Cancer Stat Facts, the relative 5-year survival rate was 68.5%, marginally lower than the survival rate observed in our study (2). Our study's marginally lower survival rate observed could be attributable to several population-specific factors, including differences in healthcare resources, screening practices, and treatment options. The study conducted by Zanoni et al. (27) between 1985 and 2015 analyzed the data of 2,085 patients with newly diagnosed oral cancer. In their retrospective cohort investigation, they found a survival rate of 64.4%, which is relatively high. In comparison, our study revealed a survival rate that was marginally lower at 50.3%. There were also statistically significant differences in our study's survival rates between clinical stages. They were 72.7% in stage I, 80% in stage II, 56.2% in stage III, and 28.1% in stage IV, with stage IV having a relatively higher mortality rate than the other stages (*P* < .001). Previous studies also had consistent results (28, 29). An American study found that oral cancer stage T4 patients had a 1.8-fold higher risk of death compared to stage T1 and a survival rate of 39.1%. However, our study showed a 2.88-fold higher risk of death and a survival rate of 29.3% when comparing the same stages (27).

Even with conventional treatments such as surgery, radiation, and chemotherapy, the prognosis and survival rate for oral squamous cell carcinoma are notoriously dismal (16, 30). However, there have been remarkable advances in the early detection of malignancies, treatment of neck lymph node metastases, postoperative chemotherapy, radiation therapy, and surgery over the past three decades, all of which have contributed to increased survival rates (31–33).

According to a study in Brazil, 77.4% of the 703 patients who were treated for oral cancer between 2007 and 2009 were male. Our research found that 63% of oral cancers were found in males, less than the Brazilian study. Comparable to the results of our study (79.2%), 73.4% of all patients were diagnosed with late-stage (III, IV) malignancies. This study discovered a 5-year survival rate of 27.9%, lower than what we discovered (50.3%). The fact that treatment alone or in combination with surgery (43.7%) was relatively low compared to surgical treatment (91.3%) likely explains why the 5-year survival rate was more than two times lower. Contrary to our findings, this Brazilian study indicates that non-surgical treatment (HR 3.11; 95%CI 2.24–4.29; p0.001) and the over-60 age group (HR 1.37; 95%CI 1.01–1.50; p0.001) were strongly associated with mortality. These variables were not associated with mortality in our study (non-surgical treatment-HR 0.83, 95% CI 0.49–1.39; p0.486, >60 years 1.52; 95% CI 0.96–2.42; p0.07). In the Brazilian study, an advanced tumor stage was associated with an increased risk of mortality (HR 2.14; 95%CI 1.68–2.74; p0.001), which was analogous to our findings (stage IV-HR 4.08; 95%CI 1.2–13.4; p0.024) (9).

Geum et al. (17) conducted a 1998–2008 study on oral cancer patients who underwent radical surgery. The 5-year survival rate was greater than our findings (50.3%) at 75.7%. According to the findings of Geum's study, in the stage I, III, and IV survival rates were higher (90%, 100%, 45.5%) than our data (72.7%, 56.3%, 28.5%), while in the stage II survival rate was comparable to our findings (80.0%). In this Korean sample study, the survival rate of patients diagnosed at stage IV was statistically different (P0.001) from the survival rate of patients diagnosed at other stages, which was consistent with our findings. In addition, the lymph node metastasis survival rate was 92.6% at N0 to 30% at N1 (P0.001), 72.8% at N0 to 40% at N1 (P0.001), and 92.6% at M0 to 0.0 at M1 0.0% (P0.001) compared to 53.3% in M0 and 0.0% (P0.001) in M1 in our study Geum et al. (17). The survival rate in N0 and M0 was greater than in our study. As tumors migrate to distant organs and lymph nodes, the metastatic process lowers the survival rate, which is consistent with the findings of our study. Even though researchers in other countries saw a greater survival rate than we did, if oral cancer is diagnosed at a late stage and has spread to other organs or lymph nodes, the patient's chances of survival fall. These studies show that there is a substantial increase in the risk of death.

Taiwanese researchers conducted a retrospective cohort analysis of 3,010 patients with oral squamous cell carcinoma who underwent surgery, radiation, and chemotherapy; 34.9% (1,050) of oral cancer sites were in the buccal portion, while 16% (482) were in the alveolar section. Oral tumors affecting the alveolar section comprised 61.2% (295) of cases, and 58.2% (92) of those affecting the retromolar space were diagnosed at an advanced stage (III, IV), whereas most other tumors were diagnosed at an early stage (I, II). However, 45.7% (79) of all malignancies in our study were in the tongue, and 13.3% (23) were in the lips, with tumors detected at a late stage in locations other than the hard palate. The location and stage of the tumors in the Taiwanese findings differed from ours. It was determined that tongue and throat cancers were more prevalent in Taiwan because of oral tobacco use (34). In Mongolia, however, cancers of the tongue and lips are prevalent, induced by alcohol and cigarette use, the sharp edges of teeth with cavities, and chronic irritation of dentures. It may be observed that the location of oral malignancies varies globally due to the factors that promote tumor growth.

According to a study conducted by Dutch researchers between 2006 and 2010, the survival rate of oral cancer patients was determined by tumor location. Tongue cancer (65%) was higher than our results (38%), gum and alveolar cancer tumors (53%), floor of the mouth tumors (57%) were the same as our results (gum and alveolar 53.3%, floor of the mouth 56.3%). The survival rate for palatal cancers was 67%, which was lower than our results (77.8%), but lip (65.2%), buccal mucosa (60%), soft palate (56.3%), and retromolar space (60%) results were higher than ours (35). A study of 6,791 cases of stage I and II oral squamous cell carcinoma diagnosed between 1998 and 2004 was done using the National Cancer Institute's (SEER) database. The results were similar to the survival rate of our findings (45.7%). The 5-year survival rate for tongue cancer was 60.4%, nearly twice as high as our findings (38%). Other oral tumors had a survival rate of 64.7%, which was the same as our findings (61.27%) (36). Because the U.S. study only included patients with early-stage oral cancer (stage I and II), the survival rate for tongue and other tumors was higher than ours. In our study, tongue cancer had a poorer survival rate than other oral malignancies (27). This is due to the tongue's biological and epidemiological differences from other parts of the oral cavity, as well as the organ's increased risk of tumor recurrence. The survivability of oral cancer varies depending on whether tumor cells enter muscles, bones, nerves, vascular tissue, metastasis to lymph nodes, or neighboring or distant organs.

Several limitations should be considered when interpreting the results of the study on prognostic factors for survival in oral cancer. These limitations include potential sources of bias and imprecision, which can affect the findings’ direction and magnitude. Selection bias may have been introduced by the study's reliance on the medical records of patients diagnosed with oral squamous cell carcinoma. It is possible that some patients were excluded from the study, such as those who sought treatment at various healthcare facilities or those whose medical records were incomplete. This could result in an inaccurate representation of the population and reduce the generalizability of the findings.

The study's design was retrospective, meaning that data was collected after the outcomes had already occurred. This increases the likelihood of recall bias and misclassification bias. Variations in the accuracy and completeness of medical records may result in imprecise or biased estimates of prognostic factors and their associations with survival outcomes. Despite adjusting for potential confounders such as age and place of residence, it is conceivable that other unmeasured or residual confounders were overlooked. The exposure (prognostic factors) and the outcome (survival) may be influenced by confounding factors, such as socioeconomic status, lifestyle factors, comorbidities, or access to healthcare. The omission of these confounding variables may result in estimations of the associations between prognostic factors and survival outcomes that are biased.

The study categorized continuous variables, such as age, which may have resulted in information loss and diminished the precision of the estimates. The choice of category boundaries may influence the interpretation of the results and may obscure or introduce artificial associations. The study's sample size was relatively small (*N* = 173), which may have limited its statistical power to detect significant associations. Small sample sizes can increase the probability of random variation and diminish the accuracy of estimates. Due to the small sample size, the magnitude of the associations reported in the study should be interpreted with caution.

Because the study focused on a specific population of patients diagnosed with oral squamous cell carcinoma, the findings may not be applicable to other populations or varieties of cancer. The study was conducted in a specific geographical area or healthcare setting, and the results may not be applicable to other settings, populations, or healthcare systems with distinct demographics or healthcare systems. So, it is crucial to consider these restrictions when interpreting the study's results. While the results provide valuable insights into the prognostic factors for oral cancer survival, the potential biases and imprecision should be considered to prevent overgeneralization and to guide future research in the field.

## Conclusion

The survival rate for oral cancer in our study was 50.3%, while the survival rate for tongue cancer was the lowest (38%). Advanced age, urban life, advanced cancer stage (IV), and cancer recurrence are all related to an increased chance of death. When compared to the outcomes of other industrialized nations, Mongolia's survival rate is relatively poor because most cancer patients are identified at a late stage. As a result, it has been established that it is vital to focus on the active surveillance of early oral cancer diagnosis among the public, primary prevention, and enhancing cancer education.

## Data Availability

The original contributions presented in the study are included in the article/Supplementary Material, further inquiries can be directed to the corresponding author.
